# Intelligent Care: A Scientometric Analysis of Artificial Intelligence in Precision Medicine

**DOI:** 10.3390/medsci13020044

**Published:** 2025-04-19

**Authors:** Khalid M. Adam, Elshazali W. Ali, Mohamed E. Elangeeb, Hytham A. Abuagla, Bahaeldin K. Elamin, Elsadig M. Ahmed, Ali M. Edris, Abubakr A. Elamin Mohamed Ahmed, Elmoiz I. Eltieb

**Affiliations:** 1 Department of Medical Laboratory Sciences, College of Applied Medical Sciences, University of Bisha, P.O. Box 255, Bisha 67714, Saudi Arabia; elshazali@ub.edu.sa (E.W.A.); melnageeb@ub.ed.sa (M.E.E.); aadlah@ub.edu.sa (H.A.A.); emfadlalla@ub.edu.sa (E.M.A.); aedris@ub.edu.sa (A.M.E.); aaelamin@ub.edu.sa (A.A.E.M.A.); elmoizie@ub.edu.sa (E.I.E.); 2 Department of Microbiology and Clinical Parasitology, College of Medicine, University of Bisha, P.O. Box 1290, Bisha 67714, Saudi Arabia; bkelamin@ub.edu.sa

**Keywords:** artificial intelligence, precision medicine, scientometric, machine learning, personalized healthcare

## Abstract

The integration of advanced computational methods into precision medicine represents a transformative advancement in healthcare, enabling highly personalized treatment strategies based on individual genetic, environmental, and lifestyle factors. These methodologies have significantly enhanced disease diagnostics, genomic analysis, and drug discovery. However, rapid expansion in this field has resulted in fragmented understandings of its evolution and persistent knowledge gaps. This study employs a scientometric approach to systematically map the research landscape, identify key contributors, and highlight emerging trends in precision medicine. **Methods:** A scientometric analysis was conducted using data retrieved from the Scopus database, covering publications from 2019 to 2024. Tools such as VOSviewer and R-bibliometrix package (version 4.3.0) were used to perform co-authorship analysis, co-citation mapping, and keyword evolution tracking. The study examined annual publication growth, citation impact, research productivity by country and institution, and thematic clustering to identify core research areas. **Results:** The analysis identified 4574 relevant publications, collectively amassing 70,474 citations. A rapid growth trajectory was observed, with a 34.3% increase in publications in 2024 alone. The United States, China, and Germany emerged as the top contributors, with Harvard Medical School, the Mayo Clinic, and Sichuan University leading in institutional productivity. Co-citation and keyword analysis revealed three primary research themes: diagnostics and medical imaging, genomic and multi-omics data integration, and personalized treatment strategies. Recent trends indicate a shift toward enhanced clinical decision support systems and precision drug discovery. **Conclusions:** Advanced computational methods are revolutionizing precision medicine, spurring increased global research collaboration and rapidly evolving methodologies. This study provides a comprehensive knowledge framework, highlighting key developments and future directions. The insights derived can inform policy decisions, funding allocations, and interdisciplinary collaborations, driving further advancements in healthcare solutions.

## 1. Introduction

The integration of artificial intelligence (AI) and precision medicine represents a transformative development in healthcare [1], offering the potential to significantly enhance individualized treatment strategies [2]. Precision medicine aims to tailor therapeutic interventions based on patients’ distinctive genetic profiles, environmental exposures, and lifestyle factors, with the overarching goal of optimizing treatment efficacy while minimizing adverse effects [3,4,5]. However, achieving such a high level of personalization necessitates the capability to process and interpret extensive, complex datasets, an area where AI technologies hold considerable promise [6]. AI encompasses a diverse array of computational methodologies, including machine learning, natural language processing, and neural networks, all of which excel in analyzing high-dimensional data with remarkable efficiency [7,8]. These advanced capabilities have catalyzed progress in precision medicine across various domains, such as genomics, medical imaging, and drug discovery [9,10]. For instance, AI-driven algorithms have demonstrated significant potential in genomic analyses by identifying disease-associated markers and predicting drug responses, thereby facilitating the development of targeted therapies. Similarly, AI applications in imaging-based diagnostics have improved disease detection and classification, achieving accuracy and efficiency levels that complement human expertise [10].

Despite these technological advancements, the rapid expansion of research at the intersection of AI and precision medicine has revealed notable challenges, including fragmented understandings of the field’s progression and persistent knowledge gaps. To address these challenges, scientometric analysis offers a systematic and quantitative framework for mapping the research landscape. This approach identifies key contributors, collaboration networks, and emerging thematic clusters, thereby shedding light on the structural and thematic evolution of this interdisciplinary domain. Tools such as VOSviewer and R-biblioshiny further enhance this process by visualizing collaborations, research hotspots, and thematic trends, offering stakeholders actionable insights into this rapidly evolving field [11].

Given the inherently interdisciplinary nature of AI and precision medicine, which combines expertise from computer science, biology, medicine, and data analytics, scientometric analysis emerges as an invaluable tool for exploring this field. By highlighting knowledge gaps and tracking emerging research trends, scientometric studies have demonstrated their utility in guiding research agendas, informing evidence-based policy, and supporting resource allocation decisions across scientific domains [12]. Scientometric approaches have been effectively used to map evolving research areas in big data, the global health system, drug induced liver injury, and precision oncology [13,14,15]; identify thematic gaps [16]; and visualize collaborative networks and knowledge structures [17,18]. These insights facilitate strategic collaborations and optimize efforts to address pressing healthcare challenges through AI-driven innovations.

This study seeks to delineate the research landscape at the convergence of AI and precision medicine through a comprehensive scientometric analysis. By examining global publication trends, identifying influential contributors, and mapping thematic clusters, it aims to provide a state-of-the-art understanding of the field’s development. The insights derived from this analysis could inform decision-making processes among researchers, policymakers, and funding agencies, fostering continued advancements in AI-assisted precision medicine and contributing to improved healthcare outcomes.

## 2. Methodology

### 2.1. Data Collection

A scientometric analysis was conducted to examine the research landscape at the intersection of precision medicine and AI. Data were retrieved from the Scopus database, selected due to its broad coverage of peer-reviewed journals, high citation tracking accuracy, and extensive indexing of scientific literature. Other databases, such as Web of Science and PubMed, were considered but not included due to their limitations in citation network analysis and coverage across disciplines.

A structured search query was designed using the following query string: ((“precision medicine” OR “personalized medicine” OR “individualized treatment) AND (“artificial intelligence” OR “AI” OR “machine learning” OR “predictive analytics”)). The Boolean operators (AND, OR, NOT) were applied to refine the search strategy and capture all relevant studies. Additionally, wildcard operators (person* and analy*) were utilized to account for variations in terminology.

The search was further refined based on the following inclusion criteria: Research field: Search restricted to titles, abstracts, and keywords. Time period: Publications from 2019 to 2024. Document type: Only articles, reviews, editorials, conference papers, letters, and conference reviews were considered. Language: Non-English documents were excluded.

**Figure 1:**
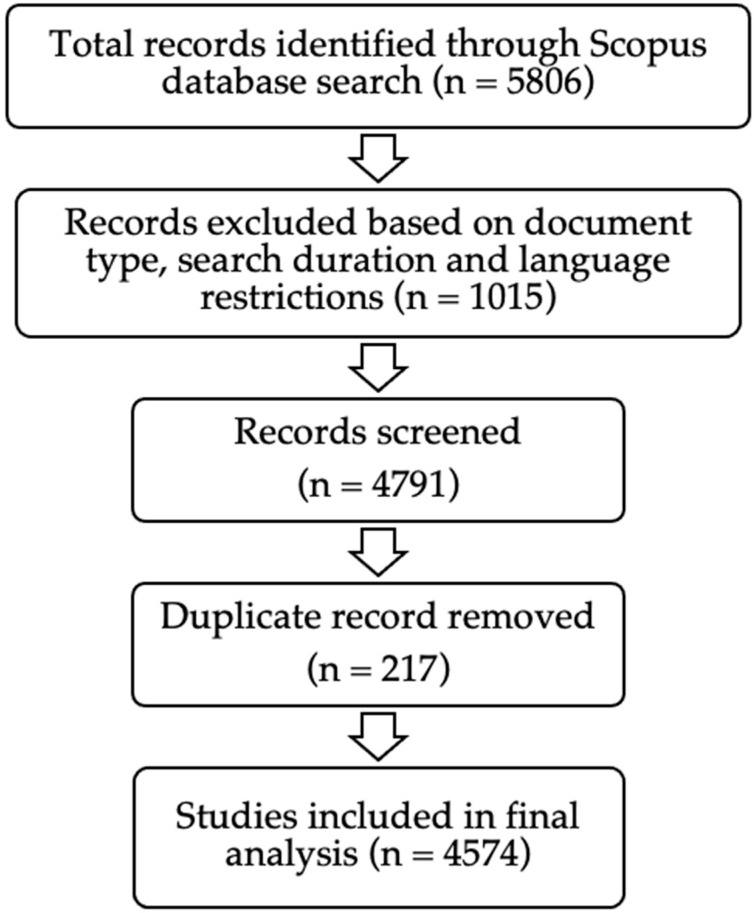
Document systematic search in Scopus database.

This process yielded an initial dataset of 5806 records, which, after applying document type and language filters, resulted in a refined dataset of 4574 records (Figure 1). The final dataset was exported in BibTeX, RIS, and CSV formats for subsequent analysis.

### 2.2. Data Processing and Normalization

The exported dataset was imported into Microsoft Excel and R-bibliometrix (biblioshiny) [19] for initial organization and preprocessing. The following data cleaning steps were performed to ensure accuracy and consistency: the removal of duplicate entries by matching DOI, title, and author names; the standardization of author names to address variations; the normalization of institutional names using OpenRefine (version 3.9.3) and manual verification; and keyword unification via the merging of synonyms and different spellings.

A manual screening of titles and abstracts was conducted to exclude irrelevant studies based on predefined conceptual criteria to ensure that only studies at the intersection of AI techniques and their application in precision medicine were included for analysis, which included the following: publications not addressing the integration of artificial intelligence (AI) with precision or personalized medicine; studies that focused mainly on traditional bioinformatics or medical imaging techniques without AI components; studies limited to general AI algorithm development without biomedical or clinical applications; and articles discussing ethical, regulatory, or educational aspects of AI in healthcare without empirical or methodological focus on precision medicine.

### 2.3. Scientometric Analysis

A multi-dimensional scientometric analysis was performed using R-bibliometrix, and VOSviewer (version 1.6.11) [20]. The analysis included the following components:

Publication trends: Annual publication output and citation trends were analyzed to assess the field’s growth over time and its scholarly impact.

Authorship and institutional productivity: Productivity and influence were evaluated using bibliometric indicators, such as the h-index and g-index. The h-index represents the number of publications (h) that have received at least h citations each, reflecting both productivity and citation impact. The g-index is an enhancement of the h-index, giving more weight to highly-cited articles by identifying the highest number (g) of papers that together received at least g^2^ citations.

Collaboration and research networks: Co-authorship analysis to assess institutional and national collaborations, co-citation analysis to identify frequently cited papers and influential references, and bibliographic coupling to explore research connections based on shared citations.

Keyword and topic analysis: Keyword co-occurrence mapping to identify research trends and emerging topics, and topic evolution mapping to examine the research focus over time.

## 3. Results

The present comprehensive scientometric analysis was conducted on the body of literature addressing AI applications in precision medicine for the period 2019–2024. During this five-year span, a total of 4574 papers were published, collectively accruing 70,474 citations. On average, each publication received approximately 15.41 citations, and the field generated an annual citation count of about 14,094.80, underscoring both the rapid dissemination and the growing scholarly impact of research in this domain.

Collaboration appears to be a hallmark of the research community in AI-driven precision medicine, as reflected by an average of 4.97 authors per paper. Additional indicators of research productivity and impact were also notable; the metrics calculated per author were 1469.43 papers and 19,247.52 citations, respectively. These figures, albeit unusually high when considered in isolation, likely reflect the aggregated contributions within extensive collaborative networks. Moreover, the scholarly influence of this field is further supported by an h-index of 110 and a g-index of 180, indicating that a substantial subset of publications has achieved a high citation threshold (Table 1).

### 3.1. Publication Trends over Time

Over the past five years (2019–2024), research at the intersection of AI and precision medicine has experienced robust growth. A scientometric analysis reveals a total of 4574 publications during this period. In 2019, the field began with 280 publications, accounting for 6.13% of the overall output. Interest in the field increased steadily, with 420 publications (9.18%) in 2020 and 604 publications (13.20%) in 2021. In 2022, there were 646 publications (14.13%), followed by a significant jump to 1055 publications (23.07%) in 2023. The upward trend culminated in 2024 with 1569 publications, representing 34.30% of the total, meaning that the last two years alone contributed over 57% of all research output in this area (Table 2).

### 3.2. Geographical Distribution and Institutional Contributions

**Figure 2:**
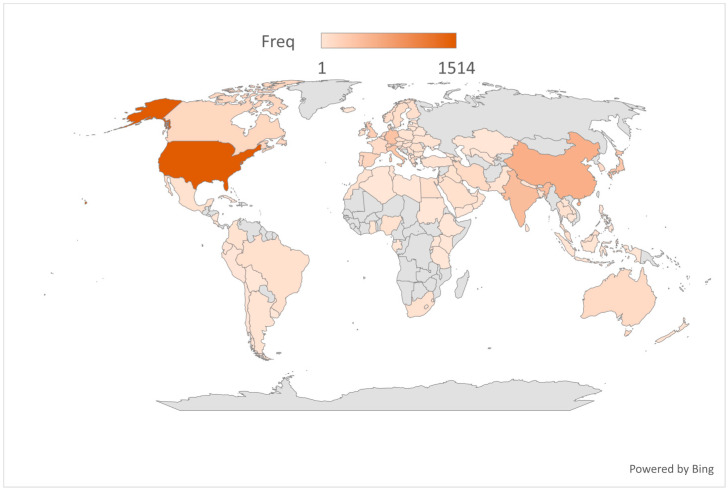
Geographical distribution of overall publications.

**Figure 3:**
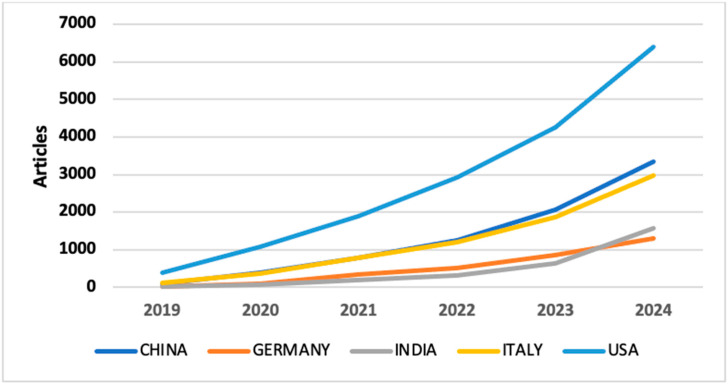
Top contributing countries overtime.

The analysis of publication output in AI-driven precision medicine reveals a substantial global contribution. The United States was the top contributor with 1514 publications, accounting for 33.1% of the total documents. China ranked second with 584 publications (12.5%), followed by India with 449 publications (9.8%), Germany with 393 publications (8.6%), and Italy with 353 publications (7.7%). Together, these five countries produced 3293 articles, representing approximately 72% of the global scientific output in the field of AI-driven precision medicine during the study period, reflecting the international interest in AI applications for precision medicine (Figure 2 and Figure 3).

**Figure 4:**
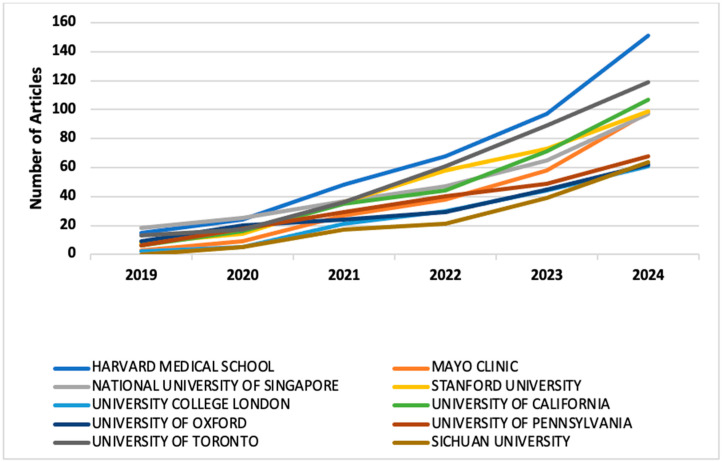
Top contributing institutions in the field over time.

Institutional analysis reveals that Harvard Medical School leads in publication output with an overall 403 publications, followed closely by the University of Toronto with overall 335 publications, the National University of Singapore with overall 289, Stanford University with overall 288 publications, the University of California with overall 279 publications, and Mayo Clinic with overall 233 publications. The University of Pennsylvania (210), University of Oxford (189 publications), University College London (163 publications), and Sichuan University (146 publications), also make notable contributions. The prominence of Harvard Medical School and the Mayo Clinic suggests their strong influence in the field, both in terms of research volume and impact (Figure 4).

**Figure 5:**
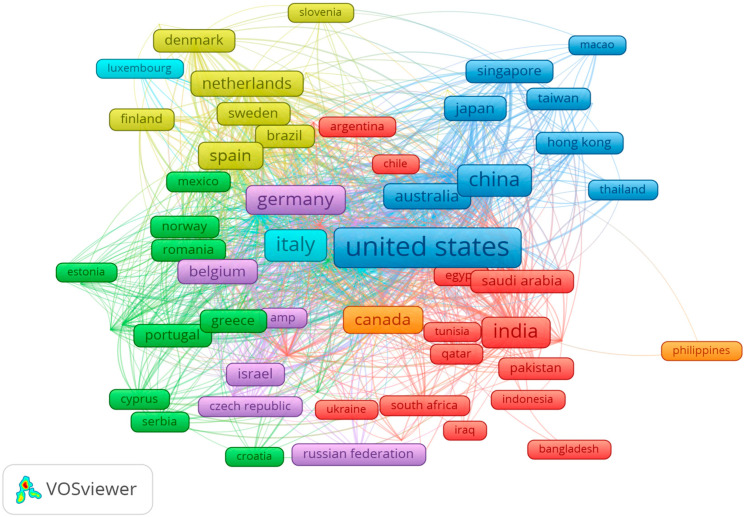
Network graph of countries collaboration.

The international co-authorship network consisted of 76 nodes (countries) and 1533 edges (collaborative links), forming 11 clusters with minimum cluster size of 2 countries, and where the size of each node reflects the total link strength, the edges represent the co-authorship ties between countries, the colors represent clusters of countries that collaborate more frequently with each other, and countries positioned closer to each other collaborate more often. The network density was 0.538 with an average degree of 40.34, indicating a highly connected global research landscape, which reflects a strong global collaboration structure in this filed. Figure 5 presents the network map of country-level collaboration. Leading institutions such as Harvard Medical School, Stanford University, and Sichuan University serve as central hubs for global research collaborations, facilitating advancements in machine learning, computational biology, and clinical AI applications (Figure 4).

### 3.3. Citation Patterns and Influential Research

**Figure 6:**
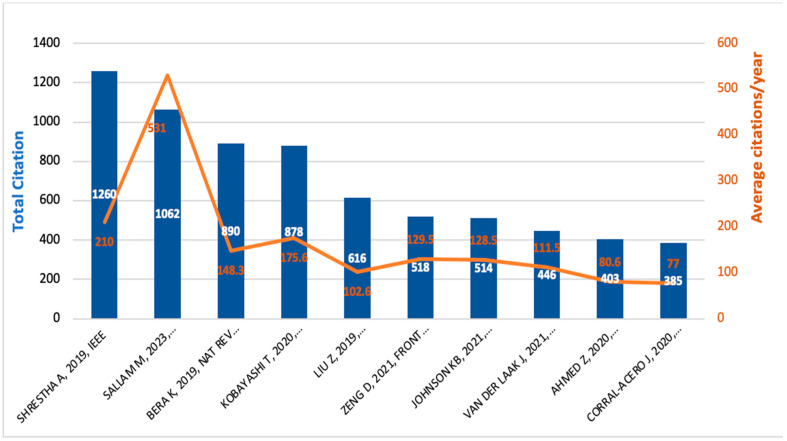
Top cited documents in terms of total citations and number of citations per year.

Figure 6 shows that among the most influential studies [21,22,23,24], Shrestha et al. [21] leads with 1260 total citations, followed by Sallam et al. [22] with 1062. Equally noteworthy are the studies by Bera et al. [23] and Kobayashi et al. [24], which have 890 and 878 citations, respectively. Looking at the annual citation rates, Sallam et al. [22] stands out by averaging 531 citations per year far exceeding its peers. In contrast, Shrestha et al. [21], despite its high overall count, averages only 210 citations per year. Other studies show a range of citation rates, underscoring the diversity of influence within the field.

### 3.4. Co-Citation Networks and Thematic Clusters

**Figure 7:**
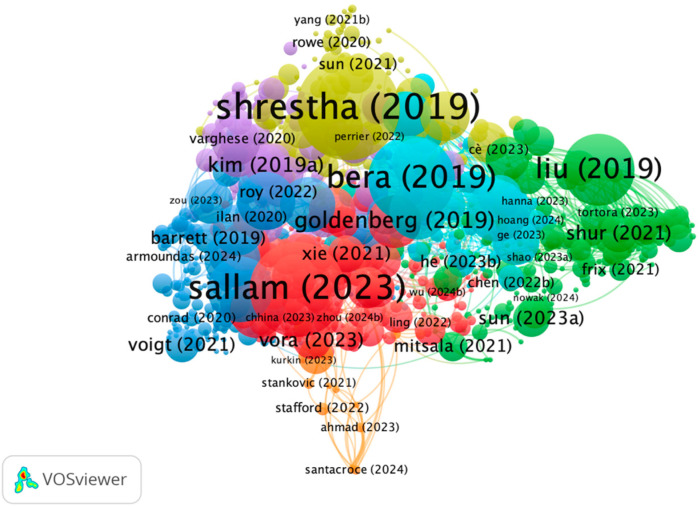
Document coupling analysis showing interconnection between publications.

Figure 7 shows the document co-citation network, the largest nodes represent the most frequently cited or interconnected publications and the color-coded clusters suggest distinct thematic and temporal groupings, where the blue cluster (2019–2021) encompasses early-stage research focusing on genomic analysis, statistical models, and AI-driven diagnostics; the yellow cluster (2020–2022) represents an intermediary phase where AI applications expanded into clinical decision-making and biomedical imaging; and the red cluster (2023–2024) is composed of newer publications that indicate the latest advancements in AI-based precision medicine, particularly in diagnosis and treatment optimization.

**Figure 8:**
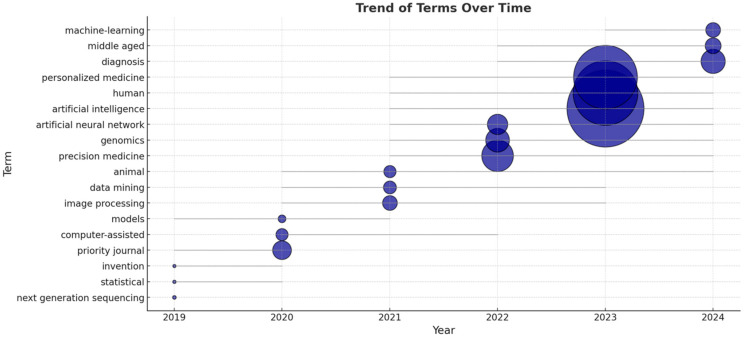
Trend of terms over time where the bubble size represents the term frequency.

Research in AI and precision medicine has evolved into distinct thematic clusters, as depicted in Figure 8. Early foundational studies [21,23,25,26], such as those by Shrestha, Liu, Bera, and Goldenberg in 2019, laid the groundwork for integrating machine learning and statistical modeling into precision medicine. These pivotal works have since been closely linked to later research that expanded into areas like genomic analysis, AI-driven diagnostics, clinical decision-making, and biomedical imaging. Over time, newer studies [27,28] have shifted focus toward advanced computational techniques, personalized medicine, and translational research.

The most recent publications [29,30,31], emerging in 2023 and 2024, highlight developments in diagnosis and treatment optimization, along with growing interests in deep learning, multi-omics data integration, and AI-based drug discovery. This progression underscores the interdisciplinary nature of the field, where collaborations across genomics, imaging, and computational diagnostics continue to drive innovative solutions in precision medicine.

### 3.5. Emerging Terms over Time

Between 2019 and 2024, research in precision medicine evolved significantly with increasing integration of AI techniques. In the early phase (2019–2020), studies [32,33] primarily focused on foundational concepts like next-generation sequencing and basic statistical methods, marking the initial integration of data-driven approaches. From 2021 onward, there was a noticeable shift as advanced AI topics [34,35] such as machine learning and artificial neural networks became more common, reflecting a broader application of AI in clinical and biomedical research. In 2022 and 2023, the emphasis further shifted toward genomics and personalized medicine, indicating a growing focus on individualized treatments. By 2024, the trend continued with a sustained interest in using AI for diagnosis and personalized treatment strategies [36,37].

Overall, three main themes emerged: the increasing reliance on AI for medical decision-making, significant advancements in genomic and precision medicine through data-driven methods, and an enhanced use of computational and image-based analysis in biomedical research (as shown in Figure 8).

## 4. Discussion

This study presents a detailed scientometric analysis of AI applications in precision medicine over the 2019–2024 period. During this interval, 4574 publications accumulated a total of 70,474 citations, reflecting both a rapid increase in research output and a substantial scholarly impact. When compared with earlier studies in similar fields [38,39,40], our analysis confirms the continued dominance of core themes such as genomic analysis and AI-driven diagnostics. For instance, Lin et al. [38] reported a compound annual growth rate of 28.4% in AI-healthcare publications from 2019 to 2023, whereas our results show a notably higher rate of 34.3% for the 2019–2024 period. This suggests a marked acceleration in scientific interest, potentially driven by post-pandemic digital transformation and increased funding for AI applications in precision medicine. The field’s dynamic growth is further underscored by an average of nearly five authors per paper, suggesting that extensive collaboration is integral to advancing research in this domain. Notably, the calculated h-index of 110 and g-index of 180 confirm that a considerable number of these studies have reached high citation thresholds, emphasizing the robust influence of this research area.

A closer examination of the publication trends reveals a marked acceleration in output over the recent years, with 2023 and 2024 alone accounting for more than 57% of the total publications. Early research (2019–2021) predominantly concentrated on genomic analysis, statistical models, and AI-driven diagnostics.

Senthil et al. [41] identified the United States, India, and China as top publishing countries, consistent with our finding that these nations contributed over 55% of total publications. However, while their study focused largely on broad AI applications in healthcare, our analysis reveals a more refined thematic clustering, especially toward personalized treatment strategies and multi-omics integration in recent years.

The strong collaboration highlighted in this study by the large network density of more than 50% is believed to enhance knowledge sharing and drive innovations in AI-driven precision medicine

As the field matured, the focus shifted towards clinical decision-making, biomedical imaging, and, more recently, the optimization of diagnosis and treatment strategies. Keyword evolution indicates a transition from foundational concepts such as next-generation sequencing and basic statistical methods to advanced topics like machine learning, deep learning, and multi-omics data integration. Highly cited studies, particularly those with significant annual citation rates like Sallam et al. [22], highlight emerging research hotspots that are likely to shape future advancements in precision medicine.

When compared with earlier studies [39,41,42,43,44,45], this study also uncovers new trends that were less apparent in prior analyses, particularly the recent surge in research addressing personalized treatment strategies and advanced computational techniques. Methodological refinements in our approach, including enhanced co-citation network mapping, have allowed for a more nuanced understanding of the evolution of research clusters over time. This comprehensive approach not only validates earlier findings but also broadens the perspective on how interdisciplinary collaborations are fostering innovation in this rapidly evolving field.

Several limitations should be acknowledged. The analysis primarily relies on data from a single major database, which may have led to the exclusion of relevant studies from other sources. Additionally, restricting the dataset to English-language publications could have overlooked significant contributions published in other languages. Although visualization tools such as VOSviewer have been instrumental in mapping thematic evolution, they may not fully capture the qualitative aspects of research impact or the subtleties of interdisciplinary collaborations. These factors should be considered when interpreting these findings.

The findings of this scientometric analysis suggest several areas for future research. Given the growing emphasis on advanced AI methodologies, there is a need to explore the integration of sophisticated computational techniques with emerging genomic and biomedical technologies. Future studies should consider integrating qualitative approaches, including expert interviews and systematic reviews, to enhance scientometric insights. In addition, funding agencies and policy makers might consider promoting international collaborations, particularly among leading institutions in the United States, China, and Europe, to further drive innovation and address the challenges of personalized medicine.

## Figures and Tables

**Table 1:** General descriptive statistics.

Item	Value
Publication years	2019–2024
Citation years	2019–2024
Papers	4574
Citations	70,474
Cites/year	14,094.80
Cites/paper	15.41
Cites/author	19,247.52
Papers/author	1469.43
Authors/paper	4.97
h-index	110
g-index	180

**Table 2:** Annual publication number and growth.

Year	Articles	% of Total	% Growth from Previous Year
2019	280	6.12%	—
2020	420	9.18%	+50%
2021	604	13.20%	+43.8%
2022	646	14.12%	+6.95%
2023	1055	23.06%	+63.3%
2024	1569	34.30%	+48.7%

## Data Availability

Data available on request from the authors.

## Abbreviations

The following abbreviations are used in this manuscript:

AI	Artificial Intelligence
CSV	Comma-Separated Values
DOI	Digital Object Identifier
RIS	Research Information System
